# B-Cell Activating Factor Increases Related to Adiposity, Insulin Resistance, and Endothelial Dysfunction in Overweight and Obese Subjects

**DOI:** 10.3390/life12050634

**Published:** 2022-04-25

**Authors:** Diana Carolina Villalpando Sánchez, Sergio Gutiérrez Castellanos, Martha Eva Viveros Sandoval, Anel Gómez García

**Affiliations:** 1 Departamento de Inmunología, Escuela Nacional de Ciencias Biológicas, Instituto Politécnico Nacional, México City 11340, Mexico; dvillalpandos1901@alumno.ipn.mx; 2 Posgrado en Inmunología, Escuela Nacional de Ciencias Biológicas, Instituto Politécnico Nacional, México City 11340, Mexico; 3 Centro de Investigación Biomédica de Michoacán, División de Investigación Clínica, Instituto Mexicano del Seguro Social, Morelia 58341, Mexico; sergio.gutierrez@umich.mx; 4 Laboratorio de Citopatología Molecular, División de Estudios de Posgrado, Facultad de Ciencias Médicas y Biológicas “Dr. Ignacio Chávez”, Universidad Michoacana de San Nicolás de Hidalgo, Morelia 58020, Mexico; 5 Laboratorio de Hemostasia y Biología Vascular, División de Estudios de Posgrado, Facultad de Ciencias Médicas y Biológicas “Dr. Ignacio Chávez”, Universidad Michoacana de San Nicolás de Hidalgo, Morelia 58020, Mexico; martha.viveros@umich.mx

**Keywords:** B-cell activating factor, obesity, insulin resistance, inflammation, endothelial dysfunction, von Willebrand factor

## Abstract

Obesity (OB) is a major healthcare problem that results from long-term energy imbalance. Adipokines and pro-inflammatory cytokines facilitate adipose tissue (AT) remodeling to safely store excess nutrients. B-cell activating factor (BAFF) is a newly described adipokine whose role in enhancing adipogenesis has been reported. The present study aimed to evaluate serum BAFF association with adiposity distribution, serum adipokines, pro-inflammatory cytokines, and metabolic and endothelial dysfunction markers. The study included 124 young Mexican adults with no diagnosed comorbidities, divided according to their BMI. Anthropometric measurements, blood counts, and serum molecules (i.e., glucose, lipid profile, insulin, leptin, pro- and anti-inflammatory cytokines, von Willebrand factor (vWF), and BAFF) were assessed. The analysis showed positive correlation between BAFF and increased fat mass in all anthropometric measurements (*p* < 0.0001). BAFF augmentation was related to systemic inflammatory environment (*p* < 0.05), and linked with insulin resistance status (*p* < 0.05). BAFF increment was also correlated with early endothelial damage markers such as vWF (*p* < 0.0001). Linear regression analysis showed a role for BAFF in predicting serum vWF concentrations (*p* < 0.01). In conclusion, our data show that BAFF is an adipokine dynamically related to OB progression, insulin resistance status, and systemic inflammatory environment, and is a predictor of soluble vWF augmentation, in young overweight and obese Mexican subjects.

## 1. Introduction

Obesity (OB) is considered “the XXI century epidemic”, as it has exponentially spread worldwide, rising over the last 40 years to affect nearly 700 million people, with an alarming increasing tendency among children and adolescents [1]. Not being exclusive to developed countries, ethnic groups, or socioeconomic strata, OB is becoming a major public health problem in Mexico, since 74% of Mexican adults are now OB or overweight (OW), while national surveys show an exponential growth, with low odds of reaching a plateau stage [2].

OB is a complex multifactorial disease derived from long-term energy imbalance between consumed and expended calories, resulting in increased fat deposits in white adipose tissue (WAT), which are detrimental to health [3]. During OB progression, WAT undergoes a dynamic remodeling process that allows safe storage of excess nutrients from high caloric intake [4]; this metabolic organ is distributed throughout the body in two major depots: subcutaneous WAT (SCAT), and visceral WAT (VAT) [5]. Though SCAT and VAT differ in their main metabolic characteristics, both are abundantly infiltrated with resident immune cells [6] that secrete pro-inflammatory cytokines such as TNF-α and IL-6, which actively promote adipocyte differentiation [7]. However, when maintained for long time, chronic low-grade inflammation has been described as one of the principal links between OB and its pathophysiological sequelae, such as insulin resistance (IR) [8], metabolic syndrome (MS) [9], endothelial dysfunction (ED) [10], type 2 diabetes mellitus (T2DM), and cardiovascular disease (CVD) [11].

Furthermore, WAT functions as an endocrine organ per se, expressing cytokines, hormones (e.g., leptin, adiponectin, resistin), and peptides termed “adipokines”, which modulate peripheral appetitive signals, as well as tissue metabolic activities [12]. B-cell activating factor (BAFF) is a newly described adipokine whose expression in mature human [13,14] and murine [15] adipocytes has been reported, as well as its role enhancing adipogenesis in vitro [16].

BAFF (also known as BLYS, TALL-1, THANK, TNFSF20, or CD257) is a cytokine member of the TNF ligand superfamily coded in the gene TNFSF13B, whose expression had traditionally been described in immune cells such as monocytes, macrophages, and dendritic cells, as well as T cells [17]. BAFF is mainly recognized by its receptor (BAFF-R), and to a lesser extent by TACI (transmembrane activator, calcium modulator, and cyclophilin ligand interactor) and BCMA (B-cell maturation antigen) receptors. BAFF-R is expressed in B cells, and activates downstream pathways that regulate cell survival and proliferation [18,19].

Thus, serum BAFF increase has been associated with autoimmune diseases in which polyclonal hypergammaglobulinemia occurs as part of the disease pathophysiology, such as systemic lupus erythematosus (SLE), Sjögren’s syndrome (SS), and rheumatoid arthritis [20]. Studies carried out in Norwegian [21] and Mexican [22] subjects with SS, as well as cohorts of Australian individuals with SLE [23], reported elevated serum BAFF concentrations. Moreover, BAFF’s role in the pathophysiology of OB, as well as its metabolic sequelae, has begun to be described. Bienertova et al. [24] reported high diet-related serum BAFF in Czech Caucasian individuals, as well as its relationship with body fat and abdominal OB in women, whereas high serum BAFF was also found to be an independent risk factor for non-alcoholic steatohepatitis (NASH) developing in OW and OB Japanese subjects [25].

Hence, serum BAFF has been related to body fat and OB-related metabolic alterations. However, studies that demonstrate BAFF’s relationship with adiposity distribution, its dynamics during OB progression, and its relationship with serum adipokines, pro-inflammatory cytokines, and metabolic and endothelial dysfunction markers, are currently lacking. Therefore, the aim of this study was to assess these features in young OW and OB Mexican adults without diagnosed comorbidities.

## 2. Materials and Methods

### 2.1. Study Group

**Figure 1:**
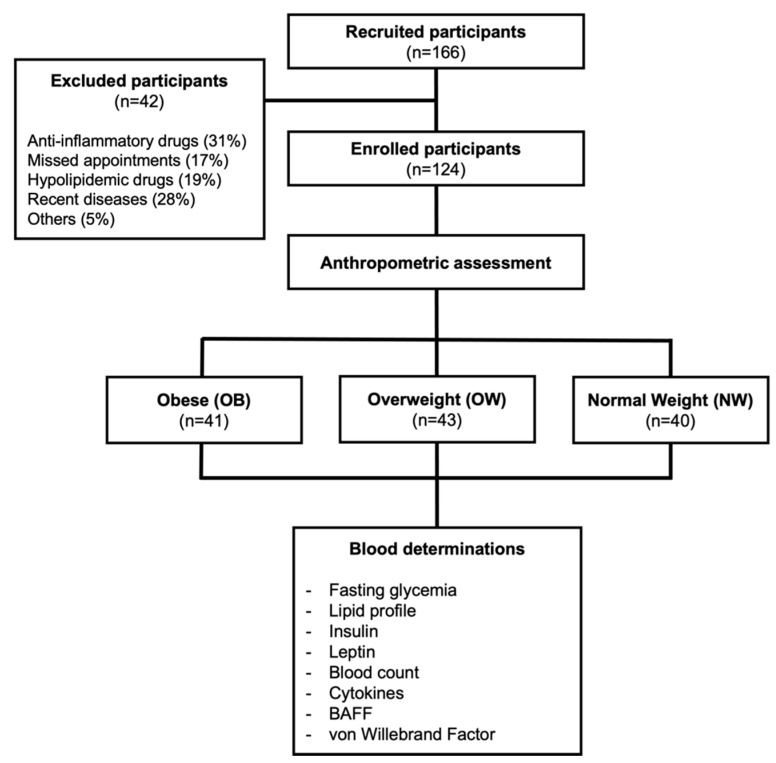
Flowchart of study participants.

A comparative cross-sectional study was carried out. This investigation was approved by the Health Research and Ethics Committee of the Mexican Social Security Institute, with the registration number R-2017-1602-7. Beneficiaries of Family Medicine Unit No. 80 of the Mexican Institute of Social Security in Michoacán, Mexico, were included. Participants were clustered in three groups based on their BMI measurements: obese (30–44.9 kg/m^2^), overweight (25–29.9 kg/m^2^), and normal weight (18.5–24.9 kg/m^2^) [26] (see study flowchart in Figure 1).

Meanwhile, sample size was calculated by a difference in proportions equation, considering an alpha significance level of 95% [27]. Participants between 18 and 55 years old, who gave their written consent and denied having been diagnosed with autoimmune diseases, immunodeficiencies, cancer, and other comorbidities (e.g., AHT, T2DM, CKD), who were not chronic consumers of alcohol or tobacco, and had not undergone invasive surgical procedures, had not suffered acute infectious or inflammatory processes (for at least a month prior to recruitment), and were not consuming antibiotic, anti-inflammatory, immunosuppressive, or immunomodulatory drugs (for at least two weeks prior to blood sampling), were included in the present study.

### 2.2. Anthropometric Measurements

Participants’ weight, height, and waist and hip circumferences were measured, as well as their percentages of body fat and lean mass, which were assessed via bioelectrical impedance analysis (BIA) (BC-558 Tanita, Arlington Heights, Illinois). Blood pressure was determined with a calibrated mercury baumanometer (W. A. Baum, Copiague, NY, USA). 

### 2.3. Hematic and Biochemical Analyses; Leucoglycemic Index Calculation

Peripheral vein puncture was performed after 12 h of fasting to examine serum molecules. Serum glycemia and lipid profile (total cholesterol, HDL-c, LDL-c, VLDL-c, triglycerides) were quantified by enzymatic–colorimetric techniques (C-4000 Architect, Abbott, Chicago, IL, USA). Complete blood counts were performed using an automated hematology analyzer (XN-1000 Sysmex, Wakinohama, Japan). Leucoglycemic index was calculated as reported by Quiroga et al. [28]: glycemia (mg/dL) × leukocyte count (10^6^/L)/1000.

### 2.4. Quantification of Serum BAFF, Insulin, Leptin, and Von Willebrand Factor

Serum BAFF (DBLYS0B Human BAFF/BLyS/TNFSF13B, R&D Systems, Minneapolis, MN, USA), insulin (EIA-2935 Insulin ELISA, DRG Diagnostics, Springfield, IL, USA), leptin (EIA-2395 Leptin Sandwich ELISA, DRG Diagnostics, Springfield, IL, USA), and von Willebrand factor (Inmubind vWF ELISA, Sekisui Diagnostics, Osaka, Japan) were assessed using commercial ELISA kits, following instructions provided by the manufacturer.

### 2.5. Quantification of Pro- and Anti-Inflammatory Cytokines

Serum cytokines (i.e., IL-2, IFN-γ, TNF-α, IL-6, IL-17A, IL-10, and IL-4) were evaluated using cytometric bead arrays (CBA Human Th1/Th2/Th17 Cytokine Kit, Becton Dickinson, Franklin Lakes, NJ, USA). According to the kit instructions, seven bead populations with distinct allophycocyanin (APC) fluorescence intensities, coated with capture antibodies specific to IL-2, IFN-γ, TNF-α, IL-6, IL-17A, IL-10, or IL-4, were mixed to form the bead array. Subsequently, each serum sample was incubated with the bead array and a mix of phycoerythrin (PE)-conjugated monoclonal antibodies specific to each assessed cytokine (i.e., IL-2, IFN-γ, TNF-α, IL-6, IL-17A, IL-10, or IL-4). A minimum amount of 3 × 10^2^ events from each bead population were acquired. Different APC fluorescence intensities were read with the FL-4 detector as indicative of each capture bead population, while PE fluorescence was directly proportional to the serum concentration of each analyzed cytokine (i.e., IL-2, IFN-γ, TNF-α, IL-6, IL-17A, IL-10, and IL-4), and was read using the FL-2 detector of a BD Accuri C6 cytometer (Becton Dickinson, Franklin Lakes, NJ, USA) (see Supplementary Figure S1).

Obtained data were analyzed using FCAP Array software (Becton Dickinson, Franklin Lakes, NY, USA), and the serum concentration (pg/mL) of each assessed cytokine was calculated via standard 4-parameter curve interpolation, as instructed in the kit.

### 2.6. Statistical Analysis

Statistical analysis was performed using SPSS version 23 and Prism 7.0 software for Mac. The Kolmogorov–Smirnov test was applied to each variable for data behavior assessment, central tendency measurements were used to summarize continuous numerical variables, and frequencies were used in categorical variables. To estimate the association between continuous numerical variables, Pearson or Spearman correlations were performed. Contrast between groups was made by mean comparison for independent-sample tests (ANOVA or the Kruskal–Wallis test), and post hoc tests were applied when needed (Tukey’s or Dunn’s multiple comparisons tests). To evaluate the relationship between a dependent variable and predictors, multiple linear regression analysis was performed. Statistical significance was denoted as * *p* < 0.05, ** *p* < 0.01, and *** *p* < 0.001.

## 3. Results

### 3.1. Characterization of the Metabolic, Inflammatory, and Endothelial Markers of Study Subjects

A total of 166 subjects were recruited to participate in the present study. Only 124 subjects met the inclusion criteria and attended their medical appointments, so they were included and classified according to their BMI: obese (OB, *n* = 41), overweight (OW, *n* = 43), or normal-weight (NW, *n* = 40) subjects, as shown in Figure 1. 

Study participants’ clinical, anthropometric, and laboratory test characteristics are shown in Table 1. Statistical differences between OB, OW, and NW subjects were observed in blood pressure (*p* < 0.0001), adiposity measurements (BMI, % body fat, visceral fat index, waist circumference, and waist–hip ratio) (*p* < 0.0001), glycemia (*p* = 0.001), triglyceridemia (*p* < 0.0001), total cholesterol, and HDL-c (*p* < 0.05). Likewise, higher levels of serum leptin, insulin, and HOMA-IR were present in OB vs. OW and NW subjects (*p* < 0.0001), while leukocyte count was increased in OB subjects (*p* < 0.05).

Nonetheless, when assessing whether these increased anthropometric and biochemical levels were beyond established limits, it was observed that only 9% of OB subjects presented high blood pressure, and 28% presented hyperglycemia, without exceeding the T2DM threshold. Moreover, dyslipidemia was the most frequent metabolic disturbance observed (50%, 26% hypertriglyceridemia; 61%, 42% high-density hypolipoproteinemia) in OB and OW individuals, respectively. Due to the aim of this research consisting of evaluating OW and OB subjects without comorbidities, the high proportion of MS (49%, 14%) and IR (89%, 47%) drew our attention (see Table 2); since at the time of recruitment none of the subjects had an established diagnosis or were on pharmacological treatment, this could have biased of results.

In that respect, when observing the occurrence of metabolic alterations in OB and OW subjects, we proceeded to characterize their systemic inflammatory status, assessing soluble molecules such as pro-inflammatory (e.g., IL-2, IFN-γ, TNF-α, IL-6, IL17A) and anti-inflammatory (e.g., IL-4, IL-10) cytokines. To determine early endothelial dysfunction status, von Willebrand factor (vWF) was measured, as this has been described as an acute-phase reactant, indicative of endothelial homeostasis disturbance [29], whereas leucoglycemic index (LGI) is a systemic inflammatory marker broadly used as a predictive parameter of poor cardiovascular prognosis in myocardial infarction [30,31], acute coronary syndrome [32], and atherothrombotic cerebral ischemia [33]. 

Inflammatory and endothelial dysfunction markers are summarized in Table 3. Statistical differences in classical OB-associated pro-inflammatory cytokines such as TNF-α (*p* = 0.001) and IL-6 (*p* = 0.019) were observed in OB vs. OW and OB vs. NW subjects. Interestingly, pro-inflammatory cytokines whose increase is less described in OB and OW subjects, such as IL-2 (*p* = 0.003) and IL-17A (*p* = 0.010), were also statistically different between these study subjects. Similarly, vWF (*p* < 0.0001) and LGI (*p* = 0.002) were found to be significantly higher in OB and OW subjects vs. NW controls, highlighting that early ED is detectable before systemic pro-inflammatory onset, which is observed until OB consolidation.

### 3.2. Serum BAFF’s Relationship with Metabolic, Inflammatory, and Endothelial Markers in the Study Subjects

**Figure 2:**
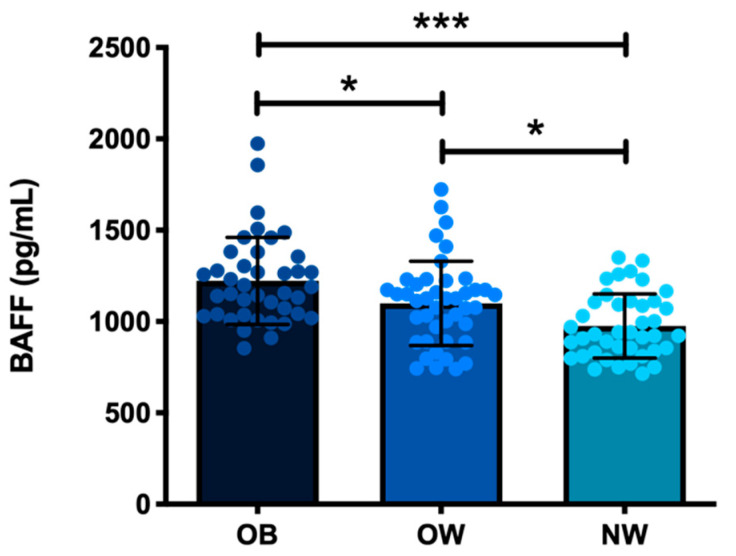
Systemic BAFF is increased in obese patients. Serum BAFF quantification was performed via ELISA. Data are shown as mean ± standard deviation. * ANOVA with Tukey’s post hoc test (*n* = 41 OB, *n* = 43 OW, *n* = 40 NW), significant differences are denoted as * *p* < 0.05, *** *p* <  0.001.

As we observed a marked pro-inflammatory systemic and ED context in subjects with increased BMI, we proceeded to quantify BAFF serum levels. We found that, similar to previous observations of metabolic, inflammatory, and endothelial affectation, serum BAFF was higher in OB (*p* < 0.0001) and OW subjects (*p* < 0.05) vs. NW controls (see Figure 2).

**Figure 3:**
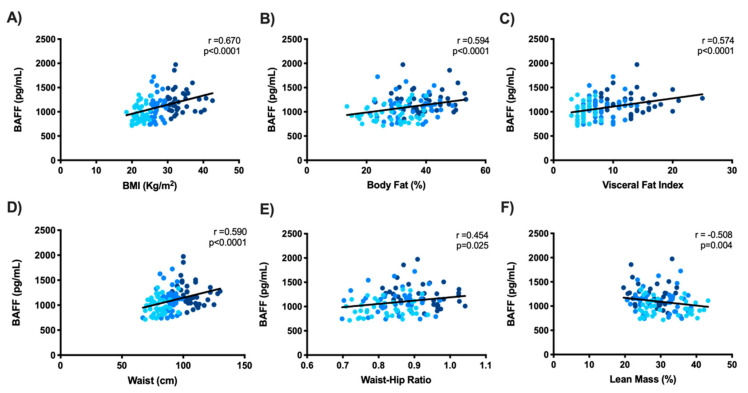
BAFF increases with body adiposity: Spearman correlation between BAFF and (**A**) BMI, and (**C**) visceral fat. Pearson correlation between BAFF and (**B**) body fat percentage, (**D**) waist circumference, (**E**) waist–hip ratio, and (**F**) lean mass percentage (*n* = 124).

As BAFF is a relatively recently described adipokine, we proceeded to assess whether its increase proportionally to BMI was directly related to any fat compartment. To do so, the relationships between BAFF and BMI, % body fat, visceral fat index (VFI), waist circumference (WC), and waist–hip ratio (WHR) were assessed, as well as the relationship between BAFF and % lean mass (see Figure 3).

No difference was observed while evaluating BAFF’s relationship with general or abdominal fat. Nevertheless, this adipokine showed strong correlation with every adiposity measurement (*p* < 0.0001), as well as a negative correlation with lean mass (*p* = 0.004), reflecting its close link with body adiposity, regardless of the anatomical region of its deposition.

**Figure 4:**
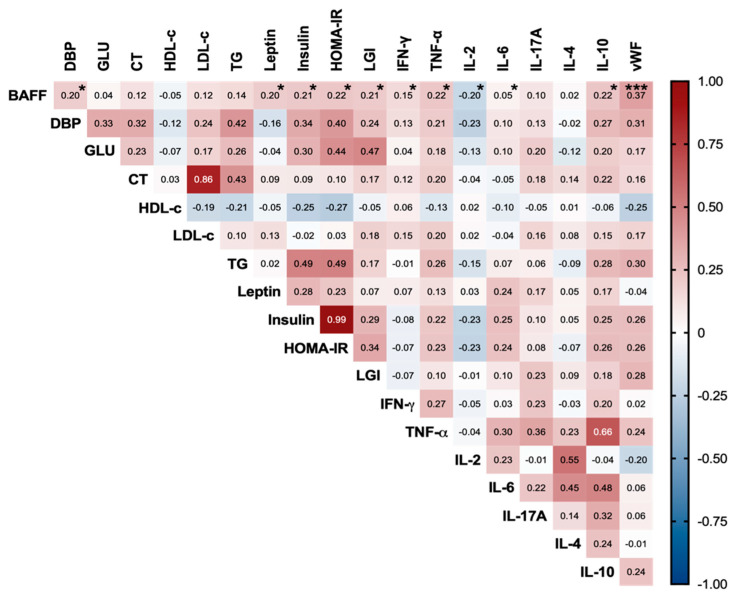
BAFF increases related to hyperinsulinemia, insulin resistance, and endothelial dysfunction. Correlogram of metabolic, inflammatory, and endothelial dysfunction markers related to obesity. Pearson and Spearman correlations are shown according to variable distribution (*n* = 124). BAFF significant correlations are denoted as * *p* < 0.05 and *** *p* < 0.001. BAFF: B-cell activating factor, DBP: diastolic blood pressure, Glu: glucose, CT: total cholesterol, HDL-c: high-density lipoprotein cholesterol, LDL-c: low-density lipoprotein cholesterol, TG: triglycerides, HOMA-IR: homeostatic model assessment, LGI: leucoglycemic index, IFN-γ: interferon gamma, TNF-α: tumor necrosis factor alpha, IL: interleukin, vWF: von Willebrand factor.

To our knowledge, there are no data on BAFF’s relationship with metabolic, serum inflammatory, and ED parameters in OW and OB subjects, so we set out to assess whether these relationships exist, and our findings are depicted in Figure 4. 

No relationship was observed between BAFF and metabolic parameters. However, a positive correlation that reached statistical significance was found between BAFF and insulinemia, as well as HOMA-IR (r = 0.2, *p* < 0.05). BAFF serum concentration also increased in a systemic inflammatory context mediated by pro-inflammatory cytokines, such as TNF-α (r = 0.22, *p* < 0.05) and IFN-γ (r = 0.15, *p* < 0.05), as well as the anti-inflammatory cytokine IL-10 (r = 0.22, *p* < 0.05).

However, it was the high magnitude of correlation between BAFF and vWF (r = 0.37, *p* < 0.0001) that caught our attention, since to the best of our knowledge no mechanistic relation between increased BAFF and ED has been reported. 

Hence, we proceeded to evaluate whether BAFF could be influencing increased serum vWF in a linear regression analysis, where classical clinical and metabolic risk factors for ED were included. Consistent with the above, the assessed model showed a significant predictive role of BAFF for increased vWF, as well as an inverse prediction of HDL-c for augmented vWF (R^2^= 0.408, F = 7.376, *p* = 0.001) (see Table 4).

## 4. Discussion

### 4.1. Low-Grade Systemic Inflammation, Insulin Resistance, and Endothelial Dysfunction in OB and OW Study Subjects

The prevalence of obesity and MS in Mexico has been consistently increasing in recent decades, mostly attributable to sedentism, high-carbohydrate and high-fat diets, genetic factors, and insufficient public policies targeted to decreasing its impact on the general population [36]. This study was conducted in a young adult population without comorbidities, despite which high dyslipidemia (hypertriglyceridemia and high-density hypolipoproteinemia), IR, and MS frequencies stood out in all study groups. Similar findings have been reported in young Mexican populations, where some of the most frequent MS traits found were low HDL-c and hypertriglyceridemia [36,37]—observations that are consistent with the high prevalence of low HDL-c levels reported in all previous Mexican health surveys [38]. Additionally, research performed in a young OW and OB Mexican population showed similar frequencies of MS and IR to those reported in this study [39]. Thus, this reflects the alarming reality of metabolic alterations in clinically healthy Mexican young adults, as well as the urgent need for corrective strategies to be applied.

In addition to metabolic alterations, during OB progression, a chronic, low-grade inflammatory state takes place [40]. Many studies have shown an increase in inflammatory mediators during OB progression, regulating different mechanisms during VAT and SCAT remodeling [41,42], among which pro-inflammatory cytokines such as TNF-α [43,44,45,46] and IL-6 [45,47,48] stand out. In this study, we observed statistically significant differences in the systemic concentrations of both pro-inflammatory (TNF-α, IL-2, IL-6, IL-17A) and anti-inflammatory (IL-10) cytokines in OB vs. NW as well as OB vs. OW subjects. There are only few reports in the literature that have evaluated this same cytokine panel using the same methodology in subjects according to their BMI; therefore, our comparison material is limited; however, Schmidt et al. [49], reported lower IL-2 and TNF-α concentrations in a study conducted in an OB German population, although it is worth noting that differences in lifestyles and genetic backgrounds could explain these differences, since our patients presented greater metabolic alterations and reported a more sedentary lifestyle than in the study of Schmidt et al. Meanwhile, in Mexican subjects, classified according to their metabolic status, Ferreira-Hermosillo et al. [50] reported serum levels of IL-6, TNF, and IL-10-α more comparable to our findings. Finally, Aradillas-García et al. [51] have recently reported a similar behavior for serum IL-17A in young OB Mexicans. Interestingly, we did not observe differences between OW and NW subjects, which allows us to highlight that, in this population, serum increase in pro-inflammatory cytokines is poorly represented during tissue remodeling and fat mass gain, consolidating until reaching OB status, and it is also noteworthy that our strict inclusion criteria allow us to attribute the inflammatory process merely to OB progression and metabolic stress, since there were no comorbidities or confounding variables in the study subjects that could bias the latter observations. 

Additionally, LGI was assessed as a reflection of inflammatory and metabolic stress, which has been used to evaluate poor prognosis in cardiovascular disease [28,31,32,33]. We found statistically significant higher LGI in OB and OW subjects, but considerably lower levels than those reported during myocardial infarction [31] and atherothrombotic cerebral ischemia [33], as was expected due to the clinical severity of those pathologies. However, LGI was expected to be higher in OB and OW subjects since, in OB progression, fatty tissue remodeling induces an adipokine profile abundant in chemoattractant molecules, as well as inflammatory ligands derived from adipocyte necrosis, hypoxia, or tissue-resident macrophage activation [52], which increase leukocyte recruitment from the bone marrow into blood circulation [53]. Though to a lesser extent, these mechanisms are importantly shared with leukocyte recruitment to the vascular endothelium during the progression of cardiovascular pathology [54].

Moreover, it has been reported that during acute [55] and chronic inflammation [56], shear forces and inflammatory molecules promote EC dysfunction, increasing vWF secretion into circulation which, upon contact with circulating platelets, can trigger thrombogenic processes [57]. In OB subjects, circulating vWF concentrations have been directly related to BMI, increasing with higher BMI of the study subjects [58,59]. Consistent with reports from the literature, we found increased vWF in OB vs. NW subjects, as well as OW vs. NW subjects. The latter is relevant, since it shows that ED starts before the onset of OB, highlighting the importance of early intervention strategies to prevent the progression of endothelial damage to cardiovascular complications in these patients.

### 4.2. Increased Serum BAFF Related to Adiposity, Insulin Resistance, Systemic Inflammation, and Endothelial Dysfunction in the Study Subjects

BAFF is a cytokine member of the TNF ligand superfamily, whose expression has been classically described in innate and adaptive immune cells [17]. More recently, BAFF’s role as an adipokine [13] was described in murine [15] and human [16] SCAT pre-adipocytes during differentiation, suggesting its pro-adipogenic role. Clinically, diet-related serum BAFF in OB Czech Caucasian subjects was reported by Bienertova et al. [24], where higher serum BAFF was observed in OB women vs. OB men. Although we did not perform gender analysis, concentrations observed in our OB group were comparable to those in OB women, while NW subjects’ BAFF was found to be slightly lower than Bienertova´s non-OB subjects; however, this could be explained as a result of their non-OB classification including 18 ≤ BMI < 30 kg/m^2^.

More recently, Chan et al. [60] conducted a study in pediatric OB patients with BMI > 50 kg/m^2^, where serum BAFF concentrations were found to be considerably lower than those observed in our OB subjects; however, concentrations of their control group were comparable to our NW group—the latter could be explained if we take into consideration that our OB subjects’ BMI was considerably lower and, to date, BAFF serum dynamics at extremely high BMI, e.g., 50 kg/m^2^, have not been fully elucidated.

Increased BAFF expression in SCAT [16] and VAT [61] has been described in high-fat diet (HFD) murine models. Meanwhile, in OB women, a positive correlation between BAFF, % body fat, and WC has been demonstrated [24]. In our study, we assessed % lean mass, % body fat, and VFI via BIA, as well as WC and WHR, to evaluate BAFF’s relationship with general and abdominal adiposity from both VAT and SCAT. Our results highlighted that serum BAFF showed an increased dynamic related to adiposity gain, regardless of body deposition. However, a limitation of the present study is that BAFF’s relationship with more SCAT deposits was not assessed to further describe this phenomenon. Additionally, we evaluated whether serum BAFF increases in OB and OW subjects were related to other adipokines, such as leptin, which increases during OB progression [12]. This relationship was reported to be negative by Bienertova et al. [24]; however, in our study’s subjects, it was found to be positive, which reflects the active adipogenic state present in these subjects.

BAFF is an adipokine that links OB to inflammation, since murine [15] and human adipocytes treated with TNF-α [13] or H_2_O_2_ [62] showed increased BAFF expression, while 3T3-L1 treatment with exogenous BAFF caused overexpression of IL-6 and TNF-α, identifying the NF-κB pathway as an effector mechanism [61]. Therefore, BAFF participates in the OB adipogenic–inflammatory axis; thus, when investigating whether there was a relationship between serum pro-inflammatory cytokines and BAFF, consistent with previous reports, we observed a positive correlation between BAFF and classically described pro-inflammatory cytokines, such as IFN-γ, TNF-α, and IL-6.

Additionally, it has been described in the literature that adipocyte treatment with exogenous BAFF decreases glucose uptake and tyrosine phosphorylation of IRS-1, inducing a cellular state of IR [61]. In the present study, we reported elevated frequencies of IR in OB (89%) and OW (47%) subjects. Interestingly we found a positive correlation between BAFF and insulinemia, as well as HOMA-IR, which mechanistically could be directly or indirectly related to systemic BAFF, through its inducing effect on pro-inflammatory cytokine expression, and their respective effects on systemic glucose metabolism.

Finally, expression of BAFF and TACI has been reported in the human vascular endothelium [16], while BAFF neutralization with anti-BAFF monoclonal antibodies in atherosclerosis-prone mice increased atherosclerotic plaque size and complexity, indicating a direct effect of BAFF on endothelial tissue [63]. In our study, BAFF was positively correlated with DPB, LGI, and vWF—a known marker of ED and atherosclerosis. Meanwhile, when constructing a linear regression model to assess whether there was influence from BAFF and classically described risk factors for ED, we found a predictive role of BAFF for serum vWF increase, so we can hypothesize that, although BAFF’s absence is detrimental to atherosclerotic progression, excessive serum BAFF—resulting from increased body adiposity and inflammation—in turn, has a detrimental effect on endothelial homeostasis, which could be indirectly mediated by TNF-α and IL-6—known endothelium activator molecules—or directly induced by BAFF downstream signaling through its TACI receptor.

## 5. Conclusions

On this basis, we conclude that in young Mexican OW and OB subjects without comorbidities, BAFF’s systemic increase is directly related to fat mass gain, in both VAT and SCAT depots. BAFF increase is related to chronic systemic inflammation and linked to IR development during OB progression. Finally, we found that BAFF influences augmented serum vWF—an early endothelial dysfunction marker. 

## Figures and Tables

**Table 1:** Clinical, anthropometric, and laboratory test characteristics of the study population.

	Obese *n* = 41	Overweight *n* = 43	Normal Weight *n* = 40	*p*
**Clinical characteristics**				
Age (years)	35.5 ± 9.0	35.2 ± 9.0	33.8 ± 9.5	0.692
SBP (mmHg)	116.5 ± 8.8 ^‡^	110.5 ± 11.5 ^ϑ^	105.1 ± 10.4	0.0001 ^Φ^
DBP (mmHg)	77.9 ± 6.4 ^‡^	73.1 ± 9.2 ^ϑ^	68.1 ± 6.6 ^θ^	0.0001 ^Φ^
**Anthropometric measurements**				
Weight (kg)	96.9 ± 15.3 ^‡^	73.7 ± 8.0 ^ϑ^	61.5 ± 7.9 ^θ^	0.0001 ^Φ^
Height (m)	1.7 ± 0.09	1.7 ± 0.08 ^ϑ^	1.7 ± 0.08	0.037 *
BMI (kg/m^2^)	33.5 ± 3.3 ^‡^	26.9 ± 1.4 ^ϑ^	22.3 ± 1.6 ^θ^	0.0001 ^Φ^
Body fat (%)	39.6 ± 7.4 ^‡^	34.4 ± 7.2 ^ϑ^	28.5 ± 7.4 ^θ^	0.0001 *
Lean mass (%)	27.3 ± 4.3 ^‡^	29.5 ± 5.3	30.1 ± 6.2	0.012 *
Visceral fat index	13.0 ± 4.5 ^‡^	8.5 ± 2.4 ^ϑ^	5.0 ± 1.3 ^θ^	0.0001 ^Φ^
Waist (cm)	106.6 ± 10.1 ^‡^	89.1 ± 5.9 ^ϑ^	79.6 ± 6.4 ^θ^	0.0001 *
WHR	0.91 ± 0.07 ^‡^	0.85 ± 0.07 ^ϑ^	0.82 ± 0.06	0.0001 *
**Biochemical parameters**				
Glucose (mg/dL)	93.7 ± 9.4 ^‡^	89.9 ± 8.5	85.9 ± 9.8	0.001 ^Φ^
Cholesterol (mg/dL)	182.0 ± 33.5	175.9 ± 32.6	164.5 ± 26.7	0.037 *
HDL-c (mg/dL)	41.7 ± 7.1 ^‡^	44.4 ± 8.4	48.9 ± 10.2	0.001 *
LDL-c (mg/dL)	110.9 ± 33.5	103.3 ± 31.2	97.8 ± 22.8	0.14
Triglycerides (mg/dL)	157.7 ± 58.5 ^‡^	140.8 ± 94.8	86.6 ± 42.5 ^θ^	0.0001 ^Φ^
**Hormones**				
Leptin (ng/mL)	11.3 ± 7.7 ^‡^	6.3 ± 7.3 ^ϑ^	7.6 ± 6.3	0.0001 ^Φ^
Insulin (µUI/mL)	27.0 ± 16.2 ^‡^	15.6 ± 11.0 ^ϑ^	8.9 ± 5.2 ^θ^	0.0001 ^Φ^
HOMA-IR	6.4 ± 4.1 ^‡^	3.6 ± 2.7 ^ϑ^	1.9 ± 1.1 ^θ^	0.0001 ^Φ^
**Hematic biometry parameters**				
Leukocytes (10^3^/µL)	7.5 ± 1.6 ^‡^	7.1 ± 1.9	6.2 ± 1.2	0.025 ^Φ^
Neutrophils (10^3^/µL)	4.2 ± 1.1	4.1 ± 1.6	3.5 ± 0.9	0.061
Lymphocytes (10^3^/µL)	2.5 ± 0.7	2.3 ± 0.7	2.1 ± 0.6	0.115
Monocytes (10^3^/µL)	0.5 ± 0.2	0.5 ± 0.2	0.4 ± 0.1	0.132
Eosinophils (10^3^/µL)	0.2 ± 0.1	0.2 ± 0.1	0.1 ± 0.1	0.072
Basophils (10^3^/µL)	0.05 ± 0.02	0.04 ± 0.01	0.04 ± 0.02	0.081
Platelets (10^3^/µL)	277.4 ± 62.5	274.5 ± 68.2	286.1 ± 72.1	0.794

SBP: systolic blood pressure, DBP: diastolic blood pressure, BMI: body mass index, WHR: waist–hip ratio, HDL-c: high-density lipoprotein cholesterol, LDL-c: low-density lipoprotein cholesterol, HOMA-IR: homeostatic model assessment. Results are displayed as the mean ± standard deviation. * ANOVA with Tukey’s post hoc test, ^Φ^ Kruskal–Wallis test, Dunn’s multiple comparisons test, ^‡^ OB vs. NW, ^ϑ^ OB vs. OW, ^θ^ OW vs. NW, *p* < 0.05.

**Table 2:** Frequency of metabolic alterations in the study population.

	Obese *n* (%)	Overweight *n* (%)	Normal Weight *n* (%)
**Fasting glucose** (≥100 mg/dL)	11 (28%)	4 (9%)	0 (0%)
**Triglycerides** (≥150 mg/dL)	20 (50%)	11 (26%)	4 (10%)
**HDL-c** (<40 mg/dL—men) (<50 mg/dL—women)	23 (61%)	18 (42%)	16 (39%)
**Blood pressure** (≥130 mmHg—systolic) (≥85 mmHg—diastolic)	3 (9%)	5 (12%)	0 (0%)
**Waist circumference** (≥102 cm—men) (≥88 cm—women)	36 (95%)	13 (31%)	3 (8%)
**Metabolic Syndrome** (>3 above criteria)	19 (49%)	6 (14%)	0 (0%)
**Insulin Resistance** (≥2.5 HOMA-IR)	37 (89%)	20 (47%)	5 (14%)

Metabolic syndrome was estimated according to Adult Treatment Panel III (ATP-III) guidelines [34], whereas HOMA-IR threshold was estimated according to literature reports [35]. HDL-c: high-density lipoprotein cholesterol, HOMA-IR: homeostatic model assessment. Results are displayed as number (frequency) of cases.

**Table 3:** Inflammatory and endothelial dysfunction markers in the study population.

	Obese *n* = 41	Overweight *n* = 43	Normal Weight *n* = 40	*p*
**Inflammatory indices**				
LGI	702.0 ±175.3 ^‡^	651.3 ± 184.5	548.3 ± 110.1 ^θ^	0.002 ^Φ^
**Pro-inflammatory cytokines**				
IFN-γ (pg/mL)	0.86 ± 0.06	0.86 ± 0.10	0.85 ± 0.07	0.217
TNF-α (pg/mL)	5.4 ± 1.0 ^‡^	4.8 ± 0.9 ^ϑ^	4.5 ± 1.0	0.001 *
IL-2 (pg/mL)	2.7 ± 1.1 ^‡^	3.1 ± 1.2	3.5 ± 1.3	0.003 ^Φ^
IL-6 (pg/mL)	5.5 ± 3.0 ^‡^	4.3 ± 1.3 ^ϑ^	6.5 ± 7.6	0.019 ^Φ^
IL-17A (pg/mL)	13.1 ± 10.3 ^‡^	9.2 ± 5.1 ^ϑ^	8.2 ± 3.1	0.010 ^Φ^
**Anti-inflammatory cytokines**				
IL-4 (pg/mL)	6.4 ± 2.2	6.4 ± 1.3	6.5 ± 1.1	0.996
IL-10 (pg/mL)	6.0 ± 4.2 ^‡^	4.7 ± 0.7 ^ϑ^	4.8 ± 1.9	0.0001 ^Φ^
**Endothelial markers**				
vWF (mU/mL)	1292.8 ± 366.1 ^‡^	1290.9 ± 435.5	527.0 ± 314.4 ^θ^	0.0001 ^Φ^

LGI: leucoglycemic index, IFN-γ: interferon gamma, TNF-α: tumor necrosis factor alpha, IL: interleukin, vWF: von Willebrand factor. Results are displayed as the mean ± standard deviation. * ANOVA with Tukey’s post hoc test, ^Φ^ Kruskal–Wallis test, Dunn’s multiple comparisons test, ^‡^ OB vs. NW, ^ϑ^ OB vs. OW, ^θ^ OW vs. NW; *p* < 0.05.

**Table 4:** Linear regression analysis of variables predicting vWF.

Variable	β	t	*p*
**Included variables**			
BAFF	0.801	2.984	0.004 **
HDL-c	−14.181	−2.409	0.018 *
**Excluded variables**			
SBP	0.086	0.774	0.441
DBP	0.080	0.717	0.476
Cholesterol	−0.007	−0.064	0.949
LDL-c	−0.022	−0.203	0.840
Triglycerides	0.027	0.247	0.806
TNF-α	0.093	0.850	0.398
IFN-γ	−0.041	−0.382	0.703
IL-6	−0.124	−1.140	0.258
LGI	0.163	1.522	0.132

BAFF: B-cell activating factor, HDL-c: high-density lipoprotein cholesterol, SBP: systolic blood pressure, DBP: diastolic blood pressure, LDL-c: low-density lipoprotein cholesterol, TNF-α: tumor necrosis factor alpha, IFN-γ: interferon gamma, LGI: leucoglycemic index, vWF: von Willebrand factor. β: coefficient β, t: t-distribution, * *p* < 0.05, ** *p* < 0.01.

## Data Availability

The data are available upon request.

## Supplementary Materials

The following supporting information can be downloaded at: https://www.mdpi.com/article/10.3390/life12050634/s1. Figure S1. Representative gating strategy of cytometric bead arrays.
