# Supplementary figures and images for: B-Cell Activating Factor Increases Related to Adiposity, Insulin Resistance, and Endothelial Dysfunction in Overweight and Obese Subjects
Source: Life (Basel). 2022 Apr 25;12(5):634. doi: 10.3390/life12050634 (PMC9146198; doi:10.3390/life12050634)

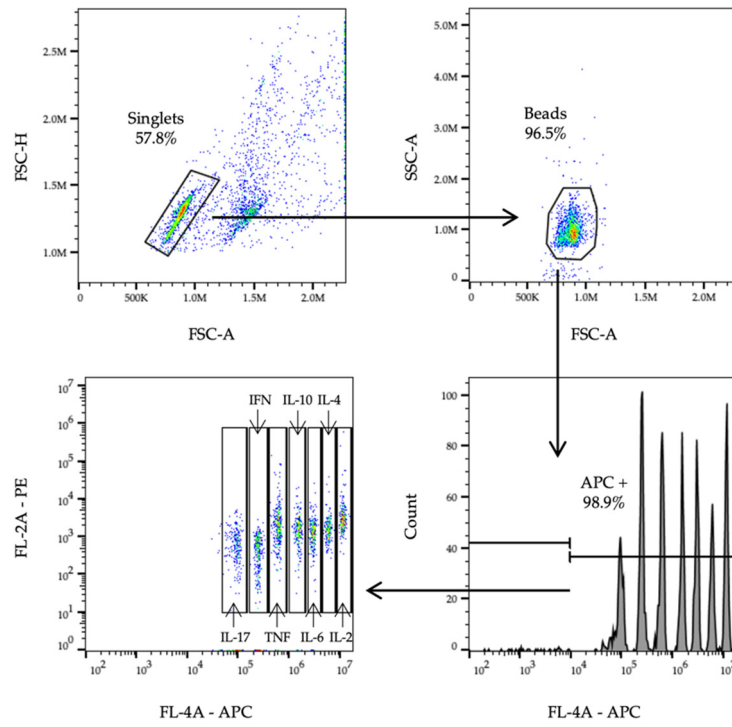

**Figure S1.** Representative gating strategy of cytometric bead arrays.

Supplement: Supplementary file 1 [file life-12-00634-s001.zip › life-1685946-supplementary.pdf]
